# The effect of backup midwife on maternal experience after vaginal childbirth – a qualitative study

**DOI:** 10.25122/jml-2021-0072

**Published:** 2022-04

**Authors:** Shiva Khodarahmi, Sepideh Hajian, Elham Zare, Malihe Nasiri

**Affiliations:** 1. Department of Midwifery, School of Nursing and Midwifery, Shahid Beheshti University of Medical Sciences, Tehran, Iran; 2. Department of Basic Sciences, School of Nursing and Midwifery, Shahid Beheshti University of Medical Sciences, Tehran, Iran

**Keywords:** midwifery, natural childbirth, experience

## Abstract

One of the goals of reproductive health enhancement is to ensure the desired experience of safe childbirth by reducing possible complications, fears, and worries about delivery by ongoing midwife backup care. This study explains women's experiences with a backup midwife during labor and childbirth. This was a qualitative study involving 19 women who had natural childbirth in Hamadan, 2020. Purposeful sampling and data collection were performed using semi-structured in-depth interviews. Data were analyzed by conventional content analysis using MAXQDA software version 10. Data analysis showed three themes and six main categories. The themes included security, high-quality care, and respectful care, consisting of two main categories of perceived empowerment and support, physiological approach and reassuring care, and respect for the mother's privacy and optimal accountability. The presence of a backup midwife during labor caused a sense of security, control, and perceived empowerment, thus a positive childbirth experience. Therefore, it is necessary to train and employ midwifery in the healthcare system. It is recommended to train and employ midwifery graduates for this purpose and include it as one of the basic principles in the current planning to promote natural childbirth.

## Introduction

Many physiological phenomena, such as pregnancy and childbirth, may be affected by pathological processes requiring health care to manage risk [1]. One of these processes is childbirth, which, in risky situations, affects self-confidence and adaptation to the role of a mother [2, 3], the start of breastfeeding [4], postpartum depression [5], and the desire to have more children [3, 6]. Simultaneously, the positive experience of natural labor is created due to women's sense of control, power, satisfaction, and confidence in childbirth. Accordingly, maintaining and promoting the mental and physiological health of the mother during pregnancy and labor is essential. This could improve women's understanding of safe childbirth as one of the influential factors in their childbirth experiences [7].

In a phenomenological study in Gonabad, Iran, women met their expectations of satisfactory childbirth in five areas, including "having a calm atmosphere without stress, being normal, acceptability of childbirth duration, feeling safe, and having control over the childbirth process" [8].

Women often do not forget their childbirth experiences; they remember the labor events fully, the conversations and actions of the care team, feeling good with help and care, or getting annoyed by their carelessness and unprofessional behavior [9].

According to two studies in Rasht on women's childbirth experience and related socio-individual parameters, the most vital factors in creating a favorable childbirth experience were the effective relationship of health care providers with the parturient and allowing her active participation in childbirth control [10], on the one hand, and improved quality of communication between women and midwifery providers and woman-centered labor care, on the other hand [11]. Also, in other studies, the presence of a reliable person accompanying the woman [12] was described as a source of psychological-emotional support in reducing maternal anxiety [13], increasing satisfaction with childbirth, and initiating timely breastfeeding [14].

Ongoing midwifery care is the best way to create a desirable experience, but also, regardless if a midwife is specialized or not, it seems to further strengthen psychological confidence and help meet the needs of labor [15]. In many cases, the lack of midwifery skills and companion knowledge during the labor process and its prolongation, especially in the first childbirth, and companion worries, can lead to fear or anxiety during the delivery, causing unnecessary interventions in the progress of natural labor. For this reason, in the principles of mother-friendly hospitals, one of the recommended measures to achieve safe childbirth is to benefit from a trained companion during labor to increase women's satisfaction with childbirth and ultimately promote maternal health. In many cases, this task is undertaken by people with academic midwifery education [16].

The presence of companions trained in western countries under the program "doula" in childbirth stages can reduce cesarean section, medical interventions such as the need for medicinal induction or invigoration in labor, and the duration of various stages of childbirth; thus, it makes women feel better about themselves [17], especially if they assist the mother since the beginning of childbirth [18, 19].

In Iran, in line with policies to promote women's health and improve the quality of hospital services, hospitals and all public and private medical centers are recommended to provide conditions for a trained companion with the mother in the delivery [20]. Since the midwife's role as a companion is the basis of midwifery care [21], it is necessary to have a backup midwife at the mother's choice from pregnancy to childbirth and after childbirth [22]. With the emphasis of national policies on population reconstruction and healthy population and promoting natural childbirth during the last decade, the need for pleasant childbirth is necessary. On the one hand, it can effectively reduce the demand for unnecessary cesarean sections, and on the other hand, it can play a vital role in transferring positive experiences from mothers to peers, thus motivating women of childbearing age to have children. However, despite implementing physiological childbirth care principles in many medical centers in the country, the importance of a backup midwife from the experiences of women who benefited from such people during childbirth has not been specifically studied. Despite various western studies showing that the backup midwife may help make labor pleasant, and according to the principles of mother-friendly hospitals announced by the Ministry of Health and Health Education of Iran to promote natural vaginal birth, utilizing backup midwives has been established only in a few hospitals – especially in private ones. Also, a very limited number of general hospitals allow the presence of backup midwives due to the internal regulations and restricted resources. Therefore, the role of these care providers on mothers' perceptions and experiences of childbirth has not been specifically studied.

The research setting of the present study is one of the few public hospitals that allow the presence of backup midwives on maternal requests in recent years. However, the mothers' experiences have not been assessed in connection with this type of support compared to the others who undertake routine care.

Therefore, due to the limited data and available studies, the present article, using a qualitative approach, aims to deeply study the role of a backup midwife in creating mothers' experiences after childbirth.

## Material and Methods

This study has a qualitative design to explore the maternal perception and experiences regarding the presence of backup midwives because, with quantitative research, we might not be able to go into the depths of the data extracted by interviews. Although the sample size is not considered as in quantitative ones, there is no precise criterion for determining the sample size until data saturation is achieved. This means that by continuing to collect data, the gathered data is a repetition of previous data, and no new information is obtained [23, 24]. So, in the present study, the adequacy of the sample size was determined based on the data saturation when no new information was discovered in the analysis.

The present study was done with the participation of 19 women referred to Fatemieh Teaching Hospital in Hamadan in 2020 for childbirth. Purposive sampling was started and continued until data saturation in the nineteenth interview (supplementing previous interviews) was achieved. Necessary information was collected using open-ended, in-depth interviews, and then they were analyzed using the conventional content analysis approach and MAXQDA software version 10.

Inclusion criteria in the study were: living in Hamadan, having a history of natural childbirth (vaginal childbirth) of a healthy single child in Fatemieh Hospital in Hamadan within the last 6 weeks of the study, having low-risk pregnancy and delivery, and the lack of evidence of medical disorder during the recent pregnancy in the mother file. Further, when the interviewer did not want to continue the interview or was not allowed to publish the quotations, the related interview was excluded from the investigation.

Sampling was performed 10 to 42 days after delivery in the obstetrics clinic of Fatemieh Hospital and three health centers, where mothers were referred for post-delivery examination and screening (baby or themselves). At first, prior to the interview, written consent was obtained from participants, and the researcher was allowed to record interviews. Furthermore, women were assured that personal information would remain confidential, and those audio files would be deleted after the interviews were conducted. 

Initially, three introductory interviews were conducted to assess the adequacy of the questions to add or modify them according to the interviewees' answers; then, the subsequent interviews continued with detailed open-ended questions. Study objectives and outcomes intended to enhance childbirth care were explained to eligible participants. The interviews were conducted in places where the mothers were comfortable, and if the participant needed to stop the interview, it was postponed to another time according to her request.

Interviews with open-ended questions began with "please describe your experience of having a backup midwife during the childbirth process". Then, they continued to explore the contributing factors to the interviewee's experiences. For cases described as vague or general, the interviewees were asked to provide further explanations using probing questions. Also, multiparous women were asked to explain, if possible, the impact of their experiences on recent and previous childbirths due to the presence of a backup midwife. The average interview time was 45 minutes.

Sampling started in May 2020 and continued until the full information saturation in November 2020 (until the 19^th^ interview).

According to the conventional content analysis, to analyze the initial data, the steps recommended by Granheim and Landman (2017) were used as below [25]:

1. Before starting the analysis, the data were transcribed. At this stage, all the visible and hidden content was written (preparation);
2. Each text of the interview was considered as a unit of analysis (definition and analysis unit);
3. Words, sentences, or paragraphs, which had relevant aspects and a single meaning in terms of content and context, were considered semantic units (semantic units);
4. Then, the text was shortened while preserving the core (summary);
5. Parts of the text related to a specific issue were identified as content domains (domain determination); 6 – A code was selected for each semantic unit (coded);
6. Groups of content with commonalities were classified as a common category (classification);
7. Finally, themes were extracted according to the hidden meanings of each category (the theme appearance).

In order to combine the data, a summary table was provided for the first participant, which was used to analyze the next cases. In the process, new themes were added to the original table, thus being progressively integrated over time to analyze the last manuscripts.

In order to ensure the accuracy and robustness of the data, Lincoln and GABA criteria were followed [26]. Accordingly, after each interview and initial coding, the meanings of the sentences were approved by each participant, and when new data were obtained, they were added to the codes. The external review method was used to increase the validity of the findings; further, the final codes and categories extracted from the quotations were independently rendered by two experts with sufficient experience in qualitative research. After discussing and exchanging views on naming some main categories or subcategories, a common and final opinion was obtained, and changes were applied. Moreover, to increase the reliability of the data, the coding-recoding method was used. Thus, immediately after the initial interview, the researcher carried out the interviews and coded the semantic units; then, two weeks later, she referred to the same analysis units again and coded them without considering the previous codes. Also, by covering a wide range of participants in terms of age, occupation, marital status, and education level, we tried to provide transferable findings for evaluation and judgment by other researchers.

## Results

In the present study, 19 interviews were performed at a maximum of 6 weeks after childbirth. The closest and farthest time intervals from childbirth to interview were 10 and 42 days, respectively. None of the interviews had to be repeated. The mean age of participants was 28.42±3.38, and the minimum and the maximum age range were 21 and 34 years, respectively. Most of the women were urban (87.65%), housewives (78.95%), and primiparous (63.16%) with a diploma and secondary education (68.42%). Some demographic and pregnancy information of the participants are presented in Table 1.

**Table 1.** Demographic and pregnancy information of participants.

**Variable**	**Number**	**Percentage**
**Parity**		
Prim parous	12	63.16
Multiparous	7	36.84
**Education**		
Primary	1	5.26
Secondary	13	68.42
Tertiary	5	26.32
**Job**		
Housewife	15	78.95
Worker	1	5.26
Employee	3	15.79
**Individual assessment of the economic situation of the family**		
Low income	1	5.26
Middle income	16	84.21
High income	2	10.53
**Infant gender**		
Female	11	57.89
Male	8	42.11
**Newborn birth weight (g)**		
2500–3500	18	94.74
More than 3500	1	5.26
**History of attending childbirth preparation classes**		
Yes	16	84.21
No	3	15.79

**Figure 1.**
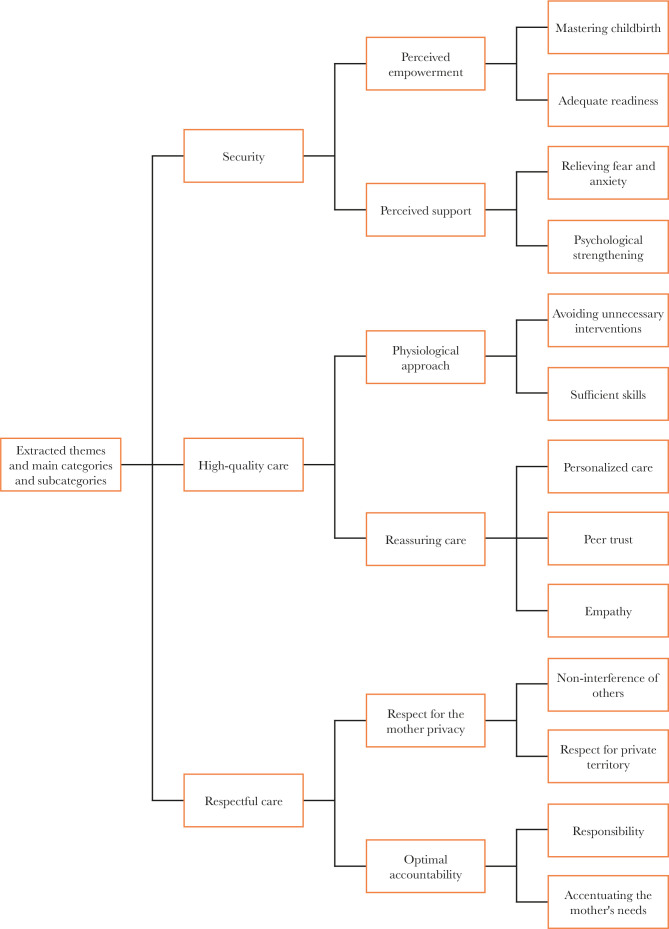
Themes, main categories, and subcategories.

At the end of data analysis and comparisons, after classifying the codes and deleting similar ones, 18 final codes, 13 subcategories, 6 main categories, and 3 themes were introduced. The most important experiences of women with backup midwives were in the themes of "security", "high-quality care", and "respectful care" (Figure 1). Examples of the initial coding process of analysis units and the process of extracting codes, categories, and themes from semantic units are shown in Table 2 and Table 3, respectively.

**Table 2.** Examples of the primary coding process of the analysis unit.

**Primary code**	**Quotation**
Good teaching of midwives	*The midwife from the class I went taught me very well what to do when I was in pain, and I knew what to do when I was in labor.*
Mother learning during pregnancy	*During pregnancy, learning from my midwife what to do helped me a lot, and I think I was in control of my delivery.*

**Table 3.** Examples of the process of extracting codes and the emergence of categories and themes from semantic units.

**Theme**	**Main category**	**Subcategory**	**Final code**	**Primary code**	**Quotation**
Security	Perceived empowerment	Mastering childbirth	Effective mother-teaching program	Good teaching of midwives	*My backup midwife in the class instructed me on what to do very well when I was in pain, and I knew what to do during labor. (P1)*
			Getting ready for childbirth	Learning during pregnancy	*During pregnancy, I learned from a backup midwife what to do during childbirth, which helped me a lot, and I think I had control over my delivery. (P2)*

### 1. Security

This theme was the result of two main categories, perceived empowerment and support, each of which included the following subcategories:

1-1 Perceived empowerment: The most common finding in participants' experiences with a backup midwife was their empowerment perception towards the delivery process, causing a desirable childbirth experience.

1-1-1 Mastering childbirth: The most significant point the interviewees mentioned was sufficient control over the process to cope with childbirth, specifically pain.

*I think, in this childbirth, by having a midwife, I could handle the delivery because I felt much better mentally.* (P8)

*Having learned from my midwife what to do when in pain helped me a lot, and I think I had control over my childbirth.* (P3)

1-1-2 Adequate readiness: A prerequisite for mothers' sense of empowerment was planning for what they would face. This was especially true for women who had previously experienced unprepared labor.

*I really was much more ready for the last childbirth than the previous one when I did not have a midwife.* (P7)

*My backup midwife taught me how to breathe during pain and pushing so that I was fully prepared for childbirth.* (P10)

1-2 Perceived support

Fear of being left alone, childbirth pain, ignorance of the childbirth process, concerns about what others say, or a less favorable experience of previous childbirth contributed to the negative emotions in mothers at the time of childbirth admission. These were all greatly reduced by a backup midwife.

1-2-1 Relieving fear and anxiety: Some participants expressed the relief of fear and anxiety by the backup midwife as a vital factor in their sense of empowerment.

*Ever since my midwife came, she made me feel less worried.* (P13)

1-2-2 Psychological strengthening: The encouragement and emotional support of the backup midwife was one of the strengthening factors mentioned by mothers in what concerns self-confidence and better feelings. 

*My backup midwife always encouraged me: you're doing so well, I'm sure you can give birth to a healthy baby soon.* (P18)

*When I was tired, the backup midwife always told me: you're one of the strongest moms I've ever seen. Thank you for your good cooperation.* (P8)

### 2. High-quality care

This main category consisted of two subcategories, physiological approach and reassuring care, which included the following subcategories:

2-1 Physiological approach: One of the aspects of a good childbirth experience from the participant's point of view was optimal care during labor.

2-1-1 Avoiding unnecessary interventions: This concept comprised excessive use of medical interventions, frequent examinations, and limiting mothers' activities during labor, which were minimized or performed according to the mothers' needs with the presence of a backup midwife. 

*My midwife did not give me any medicine to be able to give birth. She said your pain is fine and sufficient, unlike my previous childbirth, when I did not have a midwife, and as soon as I arrived at the ward, I received Pitocin (oxytocin injection).* (P16)

*Even though my room was small, my backup midwife allowed me to walk and stand, but the rest of the mothers were not allowed.* (P12)

*Thank God my backup midwife did not examine me many times. During the last birth, they examined me a lot, and I was annoyed.* (P11)

2-1-2 Sufficient skills: Some participants also mentioned having a midwife with the necessary midwifery skills as an example of quality care during labor, thus creating a good birth experience.

*I am really thankful for my backup midwife; she did venipuncture very well.* (P17)

*My backup midwife gently examined me in such a way that I was not bothered.* (P13)

2-2 Reassuring care: The mothers' perception of the midwives' essential ability to perform childbirth safely was one of the main factors in creating a desirable childbirth experience in the present study.

2-2-1 Personalized care: Receiving one-on-one care without the intervention of other caregivers was one of the characteristics of this type of care expressed by most women in this study.

*I did all my work with a backup midwife, which took much time for me.* (P8)

*All my work was done by my midwife, and she was always conscious.* (P16)

2-2-2 Peer trust: Mothers' perception of the trust of other caregivers and staff towards the backup midwife played an important role in creating and strengthening a sense of confidence in the care they received.

*I did everything with my backup midwife. I think the rest of the staff did not come to me because they were tranquil.* (P4)

*My backup midwife worked well so that the doctor and the others were confident.* (P9)

2-2-3 Empathy: The backup midwife's ability to establish an empathetic relationship with the client revealed another dimension of reassuring care.

*God bless my backup midwife; when I was in pain, she held my hand and comforted me, saying that there was nothing left to finish.* (P14)

*When I was struggling with pain, the backup midwife comforted me, saying that she knew it was hard, but it was over.* (P18).

### 3. Respectful care

This theme included two main categories, respect for mother's privacy and optimal accountability; they included the following subcategories:

3-1 Respect for the mother's privacy: Paying attention to the individual considerations and the mother's dignity during labor was one of the essential aspects of childbirth care extracted during the interviews.

3-1-1 Non-intervention of others: Several participants expressed the non-intervention of others in the presence of the backup midwife as one of its positive points.

*My backup midwife did not allow anyone to examine me except herself.* (P13)

*I looked and saw that those who did not have a backup midwife were examined by anyone who came, but for me, thank God, this was not the case.* (P6)

3-1-2 respect for private territory: Paying attention to the mother's territory, such as maintaining proper coverage during the examination and obtaining permission to perform examinations during labor, was among the other aspects of respecting the mother's privacy in labor, as mentioned by most interviewees. Having a midwife was considered an advantage compared to some previous unpleasant experiences.

*God bless the backup midwife around my bed for childbirth; she pulled the curtain during the examination.* (P19)

*My backup midwife always asked my permission before the examination, while, in my previous childbirth, staff was just asking me to lie down for examination.* (P1)

*When she (backup midwife) wanted to examine me, she closed the door to make me comfortable; this was not observed in my previous childbirth.* (P5)

3-2 Optimal accountability: The purpose was the timely presence of the backup midwife at the time of childbirth and the appropriate response and sense of responsibility to meet the physiological and psychological needs of the mother.

3-2-1 Responsibility: Having a sense of responsibility was one aspect of respectful care from the mothers' point of view. 

*My backup midwife arrived early. She arrived when I was in the emergency room.* (P11)

*For my childbirth, when my pain became severe, she (backup midwife) immediately told me to go to the hospital. I had just arrived at the hospital when she came.* (P2)

3-2-2- Emphasising the mother's needs: Paying attention to the mother's physiological needs during labor was one of the points mentioned by the interviewees as a strength.

*When I was in pain, my backup midwife massaged my back. When I was hungry, she gave me juice and dates. She did everything she could.* (P6)

*Whenever I was thirsty, my backup midwife brought me water, and whenever I was in pain, she massaged my back.* (P15).

## Discussion

The most important experience women had from a backup midwife during labor was feeling secure, resulting from the support and empowerment created by the midwife. Furthermore, women's perception of quality care given midwife's physiological approach to the childbirth process and reassuring care, combined with respectful care to maintain the values and dignity of the mother, resulted in a pleasing and satisfying experience.

The presence of a backup midwife could help the mother overcome the fear and anxiety during the childbirth process, strengthening the ability to manage the childbirth, resulting in a sense of security and a better experience. On the other hand, some mothers stated that the absence of a confident care provider, excessive examinations by staff, and the resulting pain might lead to fear and anxiety in subsequent deliveries. The trained midwife with sufficient skills was a factor in reducing this concern to improve quality care and experience by supporting, avoiding unnecessary interventions, performing personalized examinations, and empathizing with the mother.

The first theme in the present study was security. During labor, mothers need emotional and psychological support such as cuddling and comforting to dedicate themselves to the changes and ensure that everything goes well. Since security is an essential basic need, especially when perceiving fear, it can be best provided by the presence and support of a backup midwife [1].

Some studies showed fear of childbirth pain and lack of support during this process as the most important reasons women select the cesarean section [27, 28]. Psychological-emotional support from a trusted person, such as the midwife, can play an important role in feeling secure, reducing fear, and thus experiencing desirable childbirth [29]. A study in Norway in 2013 also found that building a close relationship, trust, and effective and ongoing emotional support during labor quenches the cycle of fear and stress, accelerating childbirth by creating calmness and security [30].

The second theme was high-quality care. Although high-quality service is a subjective variable that refers to people's perception of the type of care received, it is one of the ultimate goals of the health system to align to meet clients' needs [31]. Several studies showed that minimizing medical interventions during labor is associated with improving the quality of care from the perspective of women, thus resulting in their positive experience [32, 33]. Also, other studies on women's childbirth experiences indicate that they tend to have natural childbirth and that multiple medical examinations and treatments are applied as little as possible [34]. In this regard, the results of a qualitative study in Sweden showed that continuous care during childbirth by skilled midwives reduces the rate of interventions and duration of childbirth, as well as increases the quality of care, the number of normal childbirths, and mothers' satisfaction with childbirth [35].

Other studies in the United States and Canada show that continuous support during childbirth by a trained person reduces labor duration, oxytocin consumption, the number of cesarean sections and improves the quality of care and satisfying experience in mothers [36].

The third theme is respectful care. Access to the highest standard of care, including dignified and respectful care, is a fundamental right of every woman during labor [37], and respectful treatment is the most important factor in women's satisfaction with childbirth [38]. Such care is described concerning clients' privacy, avoiding ill-treatment, permission to choose, and ongoing care during childbirth [39]. According to the interviews in this study, one of the important aspects of the presence of a backup midwife is providing respectful care, which is perceived and experienced by women.

Examples of mother-centered care in various studies include factors such as respecting privacy [1], establishing a good and empathetic relationship [12], treating the mother with respect and dignity during childbirth [11], being an active listener, providing understandable information and advice to mothers, and adapting the answers to their needs [40]. Likewise, one of the most important factors in creating unpleasant experiences for women after childbirth is a situation in which emotional needs and respectful care are ignored [41]. So, it is necessary to develop respect-based care principles, like other national guidelines, to be implemented in medical centers and midwifery services systems, and adherence to these rules should be taught to students and staff with direct and indirect contact with mothers during labor.

To our knowledge, this is the first study in Iran to emphasize and explore specifically women's experiences with the presence of a backup midwife during delivery. However, despite the diversity of participants' characteristics in the present study, the findings cannot be generalized to other settings and cultures considering its qualitative approach. Moreover, some women might not have been willing to express all their feelings.

## Conclusions

The presence of a backup midwife during childbirth, by eliminating fear and anxiety, physiological approach, reassuring care, avoiding unnecessary interventions, respecting the mother's privacy, optimal accountability, and accentuating the needs of the mother led to a lasting experience of security, quality care, and respectful care in mothers.

Therefore, it is essential to train and employ some midwifery graduates for this purpose and include it as one of the basic principles in current planning in midwifery care. Also, it is recommended to offer special courses for training midwives to promote natural childbirth, avoiding unnecessary cesarean section due to fear of childbirth, thus achieving the goal of mother and infant health.

## Acknowledgments

### Conflict of interest

The authors declare no conflict of interest.

### Ethical approval

This study was approved by the Ethics Committee of Shahid Beheshti University of Medical Sciences, Tehran, Iran (IR.SBMU.PHARMACY.REC.1398.059).

### Consent to participate

Written informed consent was obtained from each participant of this study

### Personal thanks

We would like to thank the Vice-Chancellor for the Research of Hamadan University of Medical Sciences for its support and Fatemieh Hospital of Hamadan, and all the women who participated in this study.

### Authorship

SK contributed to data collection and interpretation and wrote the draft and final manuscript. SH contributed to various stages of the study design, including methodology, results interpretation, and manuscript writing. EZ and FP contributed to the development of concepts and reviewed the draft. MN contributed to data analysis and co-wrote the manuscript. All the authors read and approved the final manuscript.
